# Biaxial mechanical data of porcine atrioventricular valve leaflets

**DOI:** 10.1016/j.dib.2018.09.073

**Published:** 2018-10-03

**Authors:** Samuel Jett, Devin Laurence, Robert Kunkel, Anju R. Babu, Katherine Kramer, Ryan Baumwart, Rheal Towner, Yi Wu, Chung-Hao Lee

**Affiliations:** a School of Aerospace and Mechanical Engineering The University of Oklahoma, 865 Asp Ave., Felgar Hall Rm. 219C, Norman, OK 73019, USA; b Center for Veterinary Health Sciences Oklahoma State University, 208 S. McFarland Street, Stillwater, OK 74078, USA; c Advanced Magnetic Resonance Center, MS 60 Oklahoma Medical Research Foundation, 825 N.E. 13th Street, Oklahoma City, OK 73104, USA; d Institute for Biomedical Engineering, Science and Technology The University of Oklahoma, Norman, OK 73019, USA

## Abstract

This dataset contains the anisotropic tissue responses of porcine atrioventricular valve leaflets to force-controlled biaxial mechanical testing. The set includes the first Piola-Kirchhoff Stress and the specimen stretches (λ) in both circumferential and radial tissue directions (C and R, respectively) for the mitral valve anterior and posterior leaflets (MVAL and MVPL), and the tricuspid valve anterior, posterior, and septal leaflets (TVAL, TVPL, and TVSL) from six porcine hearts at five separate force-controlled biaxial loading protocols. This dataset is associated with a companion journal article, which can be consulted for further information about the methodology, results, and discussion of this biaxial mechanical testing (Jett et al., in press) [1].

**Specifications table**

**Table:** 

Subject area	*Mechanical Engineering*
More specific subject area	*Biomechanics of Biological Materials*
Type of data	*Text Files (.txt)*
How data was acquired	*Biaxial Mechanical Testing*
Data format	*Analyzed*
Experimental factors	*Leaflet type (MVAL, MVPL, TVAL, TVPL, TVSL), tissue direction (circumferential and radial), tissue loading rate (4.42 N/min), testing temperature (22 °C)*
Experimental features	*Employed standard biaxial testing methods to characterize the baseline anisotropic mechanical response on all porcine atrioventricular valve leaflets*
Data source location	*Norman, Oklahoma, United States*
Data accessibility	*All data mentioned in this article are contained herein.*

**Value of the data**

• Utilization for constitutive modeling of healthy atrioventricular valves.
• Comparison with material properties of diseased tissue.
• Incorporation into computational models of atrioventricular valve function.
• Reference for material properties of biomimetic tissue-engineered or bioprosthetic tissues for heart valve replacement.

## 1 Data

**Fig. 1:**
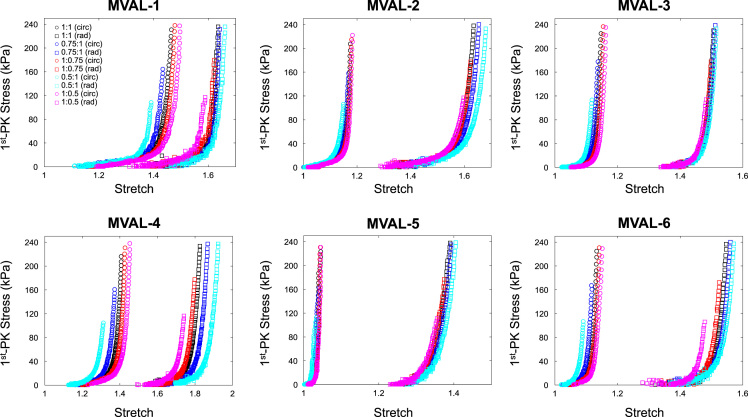
First Piola Kirchhoff stress versus stretch responses of the six MVAL tissue specimens under various biaxial mechanical loading (**P**_11_:**P**_22_ ratios of 1:1, 0.75:1, 1:0.75, 0.5:1, and 1:0.5).

**Fig. 2:**
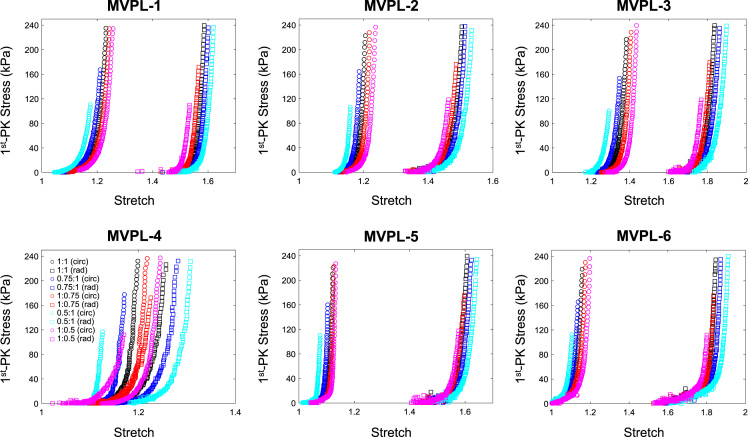
First Piola Kirchhoff stress versus stretch responses of the six MVPL tissue specimens under various biaxial mechanical loading (**P**_11_:**P**_22_ ratios of 1:1, 0.75:1, 1:0.75, 0.5:1, and 1:0.5).

**Fig. 3:**
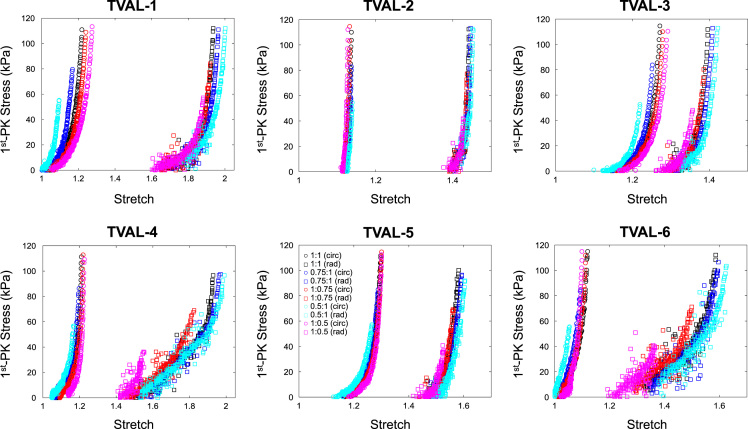
First Piola Kirchhoff stress versus stretch responses of the six TVAL tissue specimens under various biaxial mechanical loading (**P**_11_:**P**_22_ ratios of 1:1, 0.75:1, 1:0.75, 0.5:1, and 1:0.5).

**Fig. 4:**
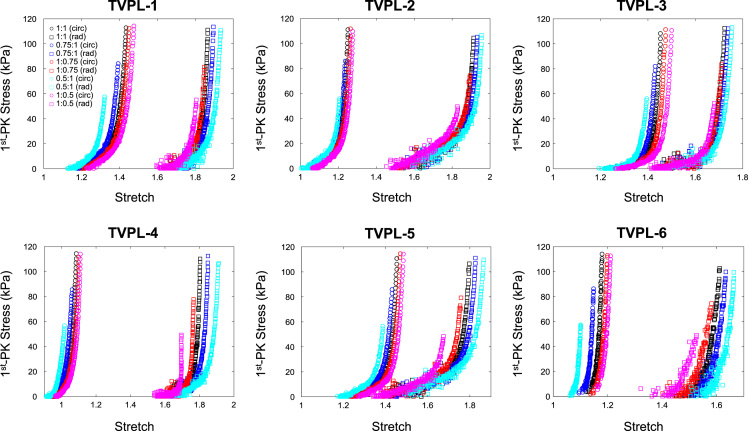
First Piola Kirchhoff stress versus stretch responses of the six TVPL tissue specimens under various biaxial mechanical loading (**P**_11_:**P**_22_ ratios of 1:1, 0.75:1, 1:0.75, 0.5:1, and 1:0.5).

**Fig. 5:**
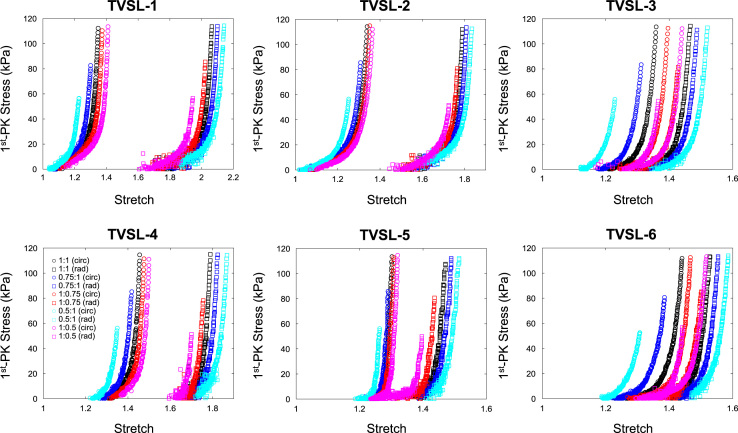
First Piola Kirchhoff stress versus stretch responses of the six TVSL tissue specimens under various biaxial mechanical loading (**P**_11_:**P**_22_ ratios of 1:1, 0.75:1, 1:0.75, 0.5:1, and 1:0.5).

The data contained in this document describes the anisotropic material response of the atrioventricular valve leaflets to biaxial mechanical loading, including mitral valve anterior leaflet (MVAL), posterior leaflet (MVPL), tricuspid valve anterior leaflet (TVAL), posterior leaflet (TVPL), and septal leaflet (TVSL). Specifically, it presents five columns of data for each test conducted. The first column represents the loading protocol, labelled as 1–5 and signifying **P**_11_:**P**_22_ ratios of 1:1, 0.75:1, 1:0.75, 0.5:1, and 1:0.5, respectively. Each test contains data from all 5 loading protocols. The maximum force applied in each test was calculated through the estimated maximum *in-vivo* stress experienced by the tissue, which was found via Laplace law for the valve during systole as 240 kPa for the mitral leaflets and 115 kPa for the tricuspid leaflets. The second and third columns display the tissue stretches in both circumferential and radial directions (*λ*_C_ and *λ*_R_, respectively), while the fourth and fifth columns are the 1st Piola Kirchhoff stresses in both circumferential and radial directions (**P**_11_ and **P**_22_, respectively). The dataset contains biaxial mechanical tests of six tissue specimens (*n* = 6) for each of the atrioventricular heart valve leaflets (MVAL: MVAL1.txt—MVAL6.txt, Fig. 1, MVPL: MVPL1.txt—MVPL6.txt, Fig. 2, TVAL: TVAL1.txt—TVAL6.txt, Fig. 3, TVPL: TVPL1.txt—TVPL6.txt, Fig. 4, and TVSL: TVSL1.txt, TVSL6.txt, Fig. 5).

## 2 Experimental design, materials and methods

### 2.1 Tissue preparation

Porcine hearts from physically healthy pigs (80–140 kg) were acquired from a local USDA approved abattoir and frozen in a standard freezer at −14 °C for storage purposes. For leaflet acquisition, the hearts were slowly thawed in a bath at room temperature, and were then dissected to obtain the atrioventricular leaflets. The leaflets were then refrigerated in phosphate buffered saline (PBS) solution at 4 °C to maintain material properties until testing.

### 2.2 Biaxial mechanical testing

For testing, an 8-mm square specimen was cut from the central region of the leaflet, and the appropriate tissue directions (circumferential and radial) were marked on the specimen. The specimen was then mounted on the commercial biaxial mechanical testing system (BioTester, CellScale, Waterloo, ON, Canada), with the tissue directions corresponding to the axes of the testing system. Briefly, four glass beads were glued onto the central region of the sample to allow optical strain tracking via digital image correlation methods with images taken from an overhead CCD camera at 15 Hz during testing. Specimens were submerged in a bath of phosphate buffered saline solution at 22 °C for the duration of the test. Eight repetitions of a force-controlled preconditioning were applied, to allow material mechanical response to converge, followed by eight cycles of each loading protocol. Applied loading data in both tissue directions were obtained from the final repetition of each loading protocol, and were combined with tissue size measurements to yield **P** in each tissue direction.

### 2.3 Tissue strain and stress calculations

Digital image correlation (DIC) based techniques have been widely utilized in the biomechanics society to track the deformations of a tissue specimen. To avoid the Saint-Venant effects on tissue deformations during our biaxial mechanical testing [2], four glass beads, served as fiducial markers, were placed in the central delimited region (3 mm × 3 mm) of the valve leaflet specimen (Fig. 2c from the companion journal article [1]). A series of images of the tissue specimen were collected by the high-resolution CCD camera and the time-dependent positions of the four fiducial markers were analyzed based on the acquired images using the DIC-based technique:

(1) $$x_{I}=X_{I}+d_{I},I=1\sim 4,$$

where **X**_*I*_׳s and **x**_*I*_׳s are the marker positions at the undeformed/reference configuration (Ω_0_) and at the deformed configuration (Ω_*t*_), respectively, and **d**_*I*_׳s are the displacement vectors of the fiducial markers, i.e., $d_{I}={\left[u_{I}(t),v_{I}(t)\right]}^{T}$.

To compute the in-plane strain of the tissue specimen, a four-node bilinear finite element was used based on the 4 markers, and the deformation gradient tensor **F** was determined using an in-house MATLAB program (R2016a, The MathWorks, Natick, MA) based on the strain-calculation technique developed previously [3], [4]:

(2) $$F=F\;(X,t)=\frac{\partial x}{\partial X}=\left[\begin{array}{cc} \sum_{I=1}^{4}B_{xI}u_{I}(t) & \sum_{I=1}^{4}B_{yI}u_{I}(t)\\ \sum_{I=1}^{4}B_{xI}v_{I}(t) & \sum_{I=1}^{4}B_{yI}u_{I}(t) \end{array}\right],$$

where *B*_*xI*_׳s and *B*_*yI*_׳s are the shape function derivatives associated with node *I* with respect to the *x* and *y* coordinates, respectively. Note that the *x*-*y* coordinates were aligned with the tissue׳s circumferential and radial directions, respectively (Figs. 1b & 2b). The right Cauchy-Green deformation tensor **C** and the Green strain tensor **E** can then be computed by

(3) $$C=F^{T}F,\;and\;E=\frac{1}{2}(C-I),$$

where **I** is the 2nd-order identity tensor. The circumferential and radial stretches, λ_C_ and λ_R_, were determined by the square roots of the principal values of **C**. Next, the first Piola-Kirchhoff (1st-PK) stress tensor **P** was computed from the applied membrane tensions, *T*_*C*_ and *T*_*R*_, as follows:

(4) $$P=\frac{1}{L\cdot t}\left[\begin{array}{cc} T_{C} & 0\\ 0 & T_{R} \end{array}\right].$$

Here, *L* is the valve leaflet specimen edge length, and *t* is the tissue thickness. For more information on the experimental procedures employed and stress and strain calculations, please refer to Section 2 and Fig. 1, Fig. 2 from the companion journal article [1].
